# PAPIMI Short Effect on Pain Perception and Heart Rate Variability in Chronic Musculoskeletal Pain: A Pilot Study

**DOI:** 10.3390/healthcare13162006

**Published:** 2025-08-15

**Authors:** Antonio Viti, Manuel Amore, Susanna Garfagnini, Diego Minciacchi, Riccardo Bravi

**Affiliations:** 1 Centro Fisioterapico Apuano, Via delle Contrade 242, 55047 Lucca, Italy; centrofisioterapicoapuano@gmail.com (A.V.); susanna1083@gmail.com (S.G.); 2 Department of Experimental and Clinical Medicine, University of Florence, Largo Brambilla 3, 50134 Florence, Italy; diego.minciacchi@unifi.it

**Keywords:** musculoskeletal pain, pain perception, heart rate variability, autonomic function, PAPIMI, pulsed electromagnetic fields, parasympathetic activity, subjective nociception, physiological adaptation

## Abstract

**Background**: Chronic musculoskeletal pain (CMP) is a multidimensional condition involving both peripheral and central mechanisms, with increasing evidence supporting an interplay between subjective pain perception and autonomic nervous system (ANS) function. However, few studies have explored whether a single non-invasive intervention can concurrently modulate both domains. **Objectives**: To evaluate the short-term effects of a single session of Pulsed Electromagnetic Field (PEMF) therapy—administered via the PAP Ion Magnetic Induction (PAPIMI™) device—on subjective pain intensity and heart rate variability (HRV) parameters in individuals with CMP. The relationship between perceived pain relief and physiological autonomic adaptations was also explored. **Methods**: Thirty adults with CMP underwent a single PAPIMI™ session. Subjective pain intensity was measured using the Numeric Pain Rating Scale (NPRS), while autonomic function was assessed via HRV. Pre- to post-intervention changes were analyzed using the Wilcoxon Signed-Rank test, while Spearman’s correlation was computed to assess associations between post-intervention changes in subjective perceived pain and HRV parameters. **Results**: A significant reduction in NPRS scores (*p* < 0.001) was found after PAPIMI intervention. Also, a significant increase in specific parasympathetic-related HRV indices, namely, RMSSD (*p* = 0.015) and HF power (*p* = 0.029), was observed. No significant correlations were found between post-intervention changes in pain perception and HRV metrics. **Conclusions**: A single PAPIMI session induced both analgesic effects and improvements in autonomic balance in individuals with CMP. These findings underscore the potential of PAPIMI as a non-pharmacological approach for rapid pain modulation and systemic rebalancing.

## 1. Introduction

Pain is a complex, multidimensional phenomenon, defined as an unpleasant sensory and emotional experience associated with, or resembling that associated with, actual or potential tissue damage [1]. Chronic musculoskeletal diseases encompass a vast spectrum of conditions affecting a substantial portion of the adult population, which provoke pain and have an impact on health status, work ability, and overall quality of life of the person [2,3,4].

A large body of experimental and clinical literature evidenced close interactions between systems involved in pain sensation and autonomic nervous system (ANS) regulation [5,6]. In this view, subjective pain perception and autonomic function may be considered as two interrelated dimensions of the same systemic dysfunction [7].

In recent years, heart rate variability (HRV) has been widely adopted as a non-invasive tool to assess ANS activity and, indirectly, to infer the organism’s health status and adaptive capacity [8,9]. Low HRV has consistently been reported in individuals with chronic musculoskeletal pain (CMP), suggesting parasympathetic withdrawal and sympathetic dominance as hallmarks of autonomic dysregulation [10,11,12]. However, although HRV provides objective physiological insight, the subjective dimension of pain remains a critical endpoint in both clinical and research settings. Pain self-report measures such as the Numeric Pain Rating Scale (NPRS) offer a valid and reliable method to quantify perceived pain intensity and reflect patient-centered outcomes [13].

While various non-pharmacological approaches have been proposed to manage musculoskeletal pain, pulsed electromagnetic fields (PEMFs) have received growing attention for their analgesic potential and autonomic effects [14,15]. Among PEMF-based devices, the PAP Ion Magnetic Induction (PAPIMI™) system stands out due to its ability to deliver high-intensity, ultra-short electromagnetic pulses without physical contact [9]. These pulses are thought to restore impaired cellular membrane potentials, enhance microcirculation, and modulate nociceptive pathways [16,17,18].

Although prior research has shown that a single PAPIMI session can induce acute increases in HRV, suggesting a parasympathetic shift, in patients with CMP [9], no studies have directly assessed its immediate effects on pain perception through validated psychometric scales. This represents a critical gap, since the modulation of pain intensity as perceived by the patient remains the principal therapeutic goal in clinical practice [19].

The aim of the present study was to evaluate the short-term effects of a single PAPIMI session on subjective pain intensity—measured using the NPRS—and autonomic function—assessed via HRV—in individuals suffering from CMP. We hypothesized that a single PAPIMI application would simultaneously reduce perceived pain intensity and increase HRV as an indicator of parasympathetic activity. Furthermore, we explored whether variations in pain scores were associated with autonomic responses, to better understand the interplay between perceived nociceptive relief and physiological adaptation.

## 2. Materials and Methods

### 2.1. Participants

Thirty individuals (20 females and 10 males) with chronic musculoskeletal pain symptoms—primarily localized in the lumbar spine (low back pain), cervical and brachial regions (cervicobrachialgia), and shoulder area (e.g., bursitis)—were enrolled for the study from the physiotherapic center “Centro Fisioterapico Apuano” in Lucca (Italy) where the study was also conducted. The mean age of the sample was 49.53 years (SD = 12.88). All individuals who were considered eligible for the study had chronic musculoskeletal pain (of more than 3 months’ duration) [2] and had not received any PAPIMI treatment before our investigation. Participants were newly admitted patients at the physiotherapy clinic and had not yet started any therapeutic intervention. Eligibility was determined during an initial clinical assessment conducted by a licensed physiotherapist, during which no treatment was provided. To minimize potential confounding, individuals who had received physiotherapy, electrotherapy, or pharmacological changes in the two weeks prior to enrollment were excluded. Exclusion criteria were the presence of cardiovascular, neurological, or psychiatric diseases; pregnancy or breastfeeding; menstrual cycle during the session; implantable electronic devices (e.g., pacemakers, defibrillators); and ring metals in the body. Moreover, participants were asked to refrain from alcohol, caffeine, and cardiovascular exercise for 24 h prior to the experimental session [9,20,21,22]. The participants’ pharmacological information was collected through an in-person interview [9]. Ongoing medications were permitted only if stable for at least two weeks prior to the intervention, in order to reduce confounding influences. Prior to the start of the study, informed consent was read and signed by each individual. The study was conducted according to the guidelines of the Declaration of Helsinki and approved by the local institutional review committee of University of Florence (Prot. N. 12774E_spe).

### 2.2. PAPIMI Intervention

**Figure 1:**
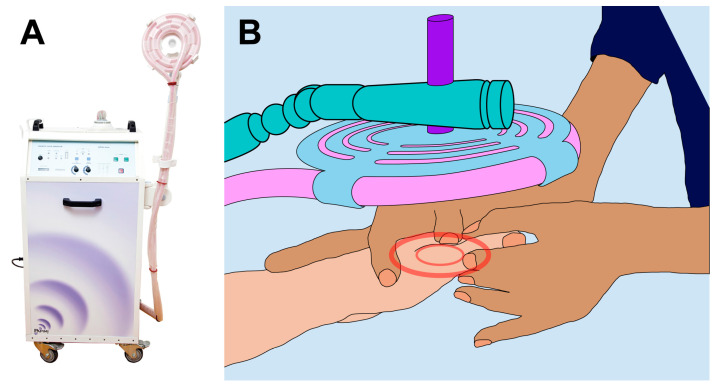
PAPIMI intervention. (**A**) PAPIMI™ device. (**B**) The physiotherapist applied the treatment coil over the referred pain zone and then adjusted the distance between the coil and the skin surface of the patient.

Pulsed electromagnetic fields treatment was performed using the PAPIMI™ device (Pulse Dynamics, model ASKLIPIOS, Athens, Greece). Manufacturer’s specifications: Input 230 V-50/60 Hz; Power 2500 VA (1.5 KW); Fuse 10 Amp, slow) (Figure 1).

Due to very high voltages reaching up to 40 kV and peak currents of several 10 kA, an energy output per pulse of about 60 W (joules) is achieved, thus emitting a magnetic induction with an intensity in the range of 50–150 mT in the vicinity of the coil applicator. Each pulse has a base frequency of about 240 kHz and a duration of about 50 µs. Although the PAPIMI system allows pulse repetition rate adjustment in the 1–8 Hz range, the frequency was set at approximately 2.5 Hz for all participants, according to the manufacturer’s clinical recommendations [23], and was not individually modified. The duration of the intervention was fixed at 30 min for each subject. The treatment was delivered once, using the circular coil applicator (diameter 18 cm), positioned approximately 4 cm from the skin, and applied over the area of reported pain.

### 2.3. Data Collection—Measurements

The primary outcome measure was subjective pain intensity perception. A written 11-point Numerical Pain Rating Scale (NPRS), one of the most commonly used scale to measure pain intensity in clinical practice, was administered to measure perceived pain intensity changes [24,25]. This pain outcome measure was shown to have excellent stability when administering over very brief periods of time (ICC > 0.90) [26,27] and high correlations with other pain measurement scales in several studies [24,27,28,29,30], making it a reliable tool for clinical assessments [31,32]. The NPRS used for this study was a 10 cm horizontal line with numbers written in ascending order from 0 to 10 from left to right, and with endpoints of 0 meaning “no pain” and 10 meaning “worst imaginable pain”. Individuals were instructed to choose a single number from the scale that best indicated their level of pain [27]. The instruction provided to each participant was: “In front of you is a numerical scale with which you will rate the intensity of your pain. On the left side is the number 0, which indicates no pain, as is written. On the right side is the number 10, which indicates the worst pain imaginable. With a pencil, circle the number representing the intensity of your current pain.”

In addition to the subjective pain intensity perception, HRV was measured as it is a valid non-invasive indicator of the state of autonomic equilibrium and provides a sensible and advanced index of the health status of the individual [33,34]. Individuals’ HRV was recorded in a seated, resting position for 5 min using a Polar H7 heart rate monitor (Polar Electro, Kempele, Finland), which consists of a single, flexible plastic sensor (2.4 × 27.9 cm) worn at the level of the xiphoid process, with the strap being wrapped around the torso by an elastic belt. During the recording session, individuals were asked to remain comfortably as still as possible and to breathe spontaneously without talking in order to not contaminate the record of HRV [9]. Pre-intervention measurements were taken immediately before the PAPIMI session. Post-intervention assessments were conducted within five minutes after the end of the 30-min treatment, to capture the acute physiological and perceptual effects of the intervention. The NPRS was administered first, followed by HRV data collection in the same environmental and procedural conditions as the baseline.

#### Offline Post-Processing of HRV Data

To calculate the HRV parameters, the recorded R-R intervals were imported and processed offline in Kubios HRV Premium 3.4.1 software [35]. R-R series of 300 cardiac beats were automatically analyzed by the software, which corrected the outliers and artifacts. If fewer than 300 R-R values were recorded, all of them were considered for the analysis. Conversely, if more than 300 R-R values were recorded, a sequence of 300 beats was selected randomly inside the period of analysis [21].

HRV assessment, based on processing recorded inter-beat intervals of the heart, was divided into time- and frequency-domain linear analysis [36]. The time-domain parameters considered in the study included the root mean square of the differences between successive pairs of R-R intervals (RMSSD), the number of successive pairs of R-R intervals that differed by more than 50 ms (NN50), the percentage of successive R-R intervals that differ by more than 50 ms (pNN50), and the standard deviation of the N-N intervals between heartbeats (SDNN). RMSSD, NN50, and pNN50 parameters are closely related to parasympathetic activity and vagal tone [9,37]. The SDNN, although influenced by both branches of the autonomic system, is predominantly mediated by the parasympathetic component in short-term recordings [9,38]. Moreover, the frequency-domain index analyzed in this study was the high frequency (HF) component between 0.15 and 0.4 Hz. The HF component reflects parasympathetic activity and is called the respiratory band because it corresponds to the HR variations related to the respiratory cycle [8,39,40,41]. High values of HF indicate a parasympathetic predominance [9,40]. The very-low-frequency band, the low-frequency band, and the LF/HF ratio were not included in the analysis as their physiological interpretation has been considered unclear [39,42,43].

### 2.4. Statistical Analysis

A Shapiro–Wilk test was performed and evidenced that all variables were not normally distributed. Data transformations were also applied, though they did not sufficiently normalize the distributions. Accordingly, non-parametric methods were used to analyze the data. The Wilcoxon Signed-Rank test was used to assess pre- to post-intervention changes in perceived pain intensity and HRV parameters. In addition, Spearman’s rank-order correlations were computed to assess associations between post-intervention changes in subjective perceived pain and HRV parameters. The delta (Δ) value for each outcome variable (HRV and NPRS) was calculated as the difference between post- and pre-intervention scores (Δ = Post − Pre) and was used to quantify the direction and magnitude of the treatment effect. Statistical significance was set at α = 0.05. The effect size was estimated using Pearson’s correlation r. According to Cohen’s recommendations for the interpretation of effect size magnitude, >0.1, >0.3, and >0.5 were considered a small, medium and large effect, respectively [44,45,46]. All analyses were conducted using IBM SPSS Statistics for Windows, Version 30.0.0 (IBM Corp., Armonk, NY, USA). A post hoc power analysis was conducted using G*Power 3.1 (Heinrich Heine University, Düsseldorf, Germany), based on the Wilcoxon signed-rank test. The expected effect size (d = 0.7) was derived from a previous randomized controlled study on the same intervention [9]. With α = 0.05, two-tailed, and a total sample size of 30, the achieved statistical power (1 − β) was 0.95.

## 3. Results

**Figure 2:**
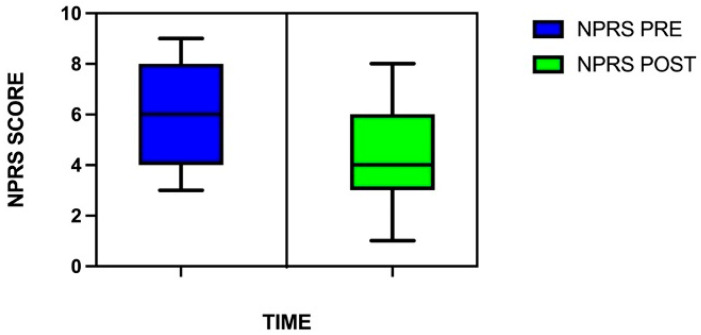
The box plot shows a statistically significant reduction in pain intensity (NPRS) from pre- to post-intervention (*p* < 0.001). This change exceeded the minimal clinically important difference (MCID) for chronic musculoskeletal pain, indicating a meaningful reduction in perceived pain. Each box represents the interquartile range (IQR), with the horizontal line indicating the median. Whiskers represent the range of values within 1.5 times the IQR. Outliers were removed for clarity but included in the statistical analysis.

A Wilcoxon Sign-Rank test was conducted to assess the effect of a single session of PAP intervention in a group of persons with chronic musculoskeletal pain. The test revealed a significant reduction in NPRS score from pre-intervention to post-intervention assessment, indicating after PAP intervention a reduction in perceived pain intensity (Table 1; Figure 2).

**Figure 3:**
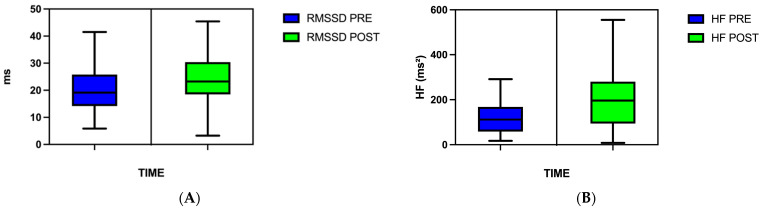
(**A**) shows significant increase in HRV time-domain RMSSD parameter from pre-intervention (blue) to post-intervention assessment (green). (**B**) shows significant increase in HRV frequency-domain HF index from pre-intervention (blue) to post-intervention assessment (green). Each box represents the interquartile range (IQR), with the central horizontal line indicating the median. Whiskers extend to the most extreme data points within 1.5 times the IQR from the lower and upper quartiles, in accordance with the Tukey method, which is widely used for visualizing non-parametric distributions. Data points outside this range (outliers) were omitted from the figures for visual clarity but were retained in all statistical analyses.

When HRV parameters were taken into account, the Wilcoxon Sign-Rank test showed a significant increase in the RMSSD index from pre-intervention to post-intervention assessment. Also, SDNN, NN50, and pNN50 evidenced trends toward an increase, though they were not significant. Moreover, a significant increase in the HF component was found after PAP intervention. Altogether, the HRV results suggested that PAP intervention induced a health status-related parasympathetic response in persons with CMP (Table 1; Figure 3A,B).

**Figure 4:**
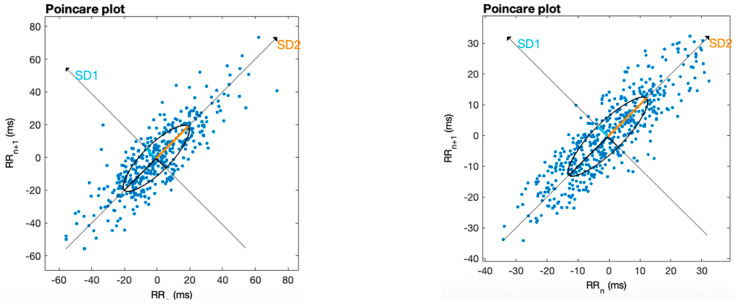
Poincaré plots for one representative participant, showing pre-intervention (**left**) and post-intervention (**right**) HRV patterns. The increased dispersion of R-R intervals post-intervention suggests enhanced autonomic complexity and vagal modulation after PAPIMI stimulation.

As a complementary visualization of the HRV dynamics, Poincaré plots were generated for one representative individual (see Figure 4). Figure 4 shows the Poincaré diagrams before and after the intervention for this representative case of the entire sample. These plots visually illustrate the increased dispersion of R-R intervals following the PAPIMI intervention, indicating enhanced autonomic flexibility and parasympathetic modulation [47].

Finally, Spearman’s rank-order correlations were finally performed to explore the relationship between post-intervention changes in subjective perceived pain intensity and HRV parameters. The test showed no significant correlations between ΔNPRS and ΔRMSSD or ΔHF, respectively.

## 4. Discussion

The present study aimed to explore the short effect of a single session of Pulsed Electromagnetic Field (PEMF) therapy, administered via the PAP Ion Magnetic Induction (PAPIMI™) device, on subjective pain intensity and HRV parameters in individuals with CMP. The intervention led to a significant decrease in NPRS scores, indicating a reduction in pain intensity, as well as a moderately significant increase in specific parasympathetic-related HRV indices, namely, RMSSD and HF power. However, no correlation between subjective pain intensity reduction and HRV increase was found significant.

A decrease in pain intensity was found after the treatment. This effect was large in magnitude (r = −0.9), underscoring the potential of PAPIMI therapy to produce meaningful short-term decrease in subjective pain perception. Importantly, the observed 2-point median reduction in the NPRS also meets the minimal clinically important difference (MCID) threshold for chronic musculoskeletal pain, as proposed by a previous study [48]. This indicates that the improvement in pain was not only statistically significant but also clinically meaningful from the patient’s perspective [48]. While much of the literature on PEMFs for chronic pain involves multi-session protocols, the current results suggest that even a single PAPIMI session can elicit rapid analgesic effects. These findings are consistent with prior works demonstrating significant effects of PEMF therapy in conditions such as low back pain [49] and fibromyalgia [50]. However, it is important to note that those studies employed lower-intensity devices—specifically, a low-frequency PEMF system [49] and a transcranial low-intensity device [50]—which differ substantially from high-intensity PEMF PAPIMI device. Therefore, while the outcomes are directionally consistent, the underlying mechanisms and stimulation parameters may not be directly comparable. Notably, more recent studies have begun to explore the clinical utility of high-intensity PEMF stimulation. For example, Viti et al. (2023) [9] demonstrated significant autonomic effects (i.e., increased parasympathetic activity) following a single PAPIMI session in individuals with chronic musculoskeletal pain. Furthermore, Keilani et al. (2025) [51] reported promising results regarding the feasibility and physiological effects of high-intensity pulsed magnetic field therapy in patients with post-COVID-19 fatigue syndrome, further supporting the systemic modulatory potential of these interventions. These findings suggest that high-intensity PEMF devices like PAPIMI may exert broader biological effects beyond local analgesia, warranting further investigation in musculoskeletal pain contexts.

One possible mechanism underlying the rapid reduction in pain perception may be related to the bioelectromagnetic interactions at the cellular level [16,17]. Chronic pain has been associated with mitochondrial dysfunction leading to impaired microcirculation, oxidative stress, and altered neuronal membrane potentials, all of which may contribute to peripheral and central sensitization [52]. Reduced oxygen availability and cellular energy imbalance may compromise the membrane potential—typically around −70 to −75 mV in healthy cells—disrupting ion exchange and cellular signaling [18,53].

PEMFs, particularly those delivered through high-intensity and short-duration bursts like those of the PAPIMI device, have been shown to induce weak microcurrents in body tissues. These may influence ion dynamics across the cell membrane, potentially facilitating the restoration of physiological membrane potentials. This bioelectrical modulation may, in turn, enhance local cellular function and tissue homeostasis [16,17,18,54].

Importantly, the restoration of ionic balance and microvascular regulation in the affected area could attenuate nociceptive signaling by reducing local inflammation, or neural hyperexcitability—mechanisms commonly implicated in chronic pain maintenance [55,56]. Such peripheral changes may also modulate the activity of afferent sensory pathways and thereby influence central pain processing.

This local rebalancing may further manifest systemically through increased parasympathetic activity [10,11,39,57,58], as evidenced by the increase in HRV indices observed in the present study. Although still hypothetical, this interpretation aligns with our previous observations [9] and is supported by literature suggesting that improvements in peripheral tissue status can feed back to central autonomic and pain-modulating networks [59].

These mechanisms suggest that the effects of PAPIMI may not be limited to neural modulation alone but instead reflect a rapid, multisystemic biological response capable of producing analgesia even after a single application.

In addition to pain relief, the study revealed a statistically significant increase in HRV parameters associated with parasympathetic activity, namely, RMSSD and HF power. These measures are considered reliable indicators of vagal tone and autonomic flexibility [8,33]. A recent systematic review of HRV in chronic musculoskeletal pain supports these findings, reporting lower parasympathetic-related indices such as RMSSD and HF in clinical populations compared to healthy controls [12]. The observed increases in these parameters support the hypothesis that PAPIMI therapy may acutely enhance parasympathetic nervous system activation, potentially fostering a relaxation response that complements pain reduction. These findings are consistent with prior research indicating that manipulative therapies and PEMF can increase vagal output in both healthy and clinical populations [9,21].

Interestingly, no significant correlations were found between changes in HRV parameters and reductions in pain intensity. While HRV provides objective insight into autonomic function, it does not always correspond linearly with subjective pain reports. This dissociation between the physiological and perceptual domains has already been observed [60] and is likely due to the fact that pain perception is shaped by multiple, interacting components—affective, cognitive, motivational, and contextual [61,62].

Pain is now widely recognized as a complex construct, shaped by individual expectations, emotional regulation, memory, and attentional focus [63,64]. Even in the presence of physiological improvements—such as increased parasympathetic activity indexed by HRV—patients may not report equivalent reductions in pain intensity [60].

This apparent dissociation should not be interpreted as a lack of interaction but rather as evidence of a complex and non-linear relationship between subjective pain and physiological adaptation [65]. HRV might reflect the organism’s general adaptive capacity or resilience [57], which supports but does not fully determine the conscious experience of pain. Likewise, reductions in perceived pain may involve shifts in brain circuits related to attention, emotion, or self-regulation, independent of peripheral autonomic changes [64,65].

In this light, our findings underscore the need to consider subjective nociceptive relief and physiological adaptation as complementary yet partially dissociable facets of a broader systemic response to treatment. Better understanding the interplay between these two domains may guide the development of more integrative pain management strategies, capable of targeting both peripheral-autonomic and central-perceptual processes.

Altogether, the present study provides preliminary evidence that a single session of PAP Ion Magnetic Induction (PAPIMI™) therapy may elicit a beneficial physiological response in individuals with chronic musculoskeletal pain, as reflected by both enhanced autonomic function and reduced subjective pain perception.

### 4.1. Strengths and Limitations

This study presents several strengths. It employed validated and widely accepted outcome measures (NPRS and HRV indices), used standardized environmental and procedural conditions during data collection, and targeted a real-world clinical population with chronic musculoskeletal pain. Furthermore, it explored the short-term response of both subjective pain intensity and objective autonomic function following a single session of high-intensity PEMF therapy via the PAPIMI™ device—an approach still underexplored in the current literature.

However, several important limitations must be acknowledged. Most notably, the absence of a sham or control group limits the internal validity of the findings and the ability to draw causal inferences—particularly for subjective outcomes, which are inherently susceptible to expectation bias and placebo-related mechanisms. Although previous randomized controlled trials have demonstrated that sham PAPIMI stimulation does not induce significant changes in autonomic parameters, the present study investigated both physiological and perceptual outcomes in a new cohort and context. Therefore, the possibility that the observed effects—especially the reduction in perceived pain—may reflect spontaneous variation, regression to the mean, or non-specific influences cannot be ruled out. Additionally, the single-session design does not allow for the evaluation of cumulative or sustained effects over time. The sample size, while adequate for detecting within-subject differences, remains relatively small, limiting generalizability. Furthermore, the lack of participant and assessor blinding, as well as the exclusion of certain commonly reported HRV indices (e.g., LF/HF ratio), may restrict comparability with existing studies. As such, the results should be interpreted as exploratory and hypothesis-generating rather than confirmatory. Future studies employing randomized, controlled, and blinded designs are warranted to validate these preliminary findings and to better delineate the specific contribution of PAPIMI stimulation to both pain relief and autonomic modulation.

### 4.2. Practical Applications

From a clinical perspective, the findings of this pilot study suggest that PAPIMI therapy may serve as a rapid, non-invasive tool for the short-term modulation of pain perception and autonomic balance in patients with chronic musculoskeletal conditions. The intervention was well tolerated, required minimal setup, and produced measurable changes in just 30 min, making it suitable for integration into physiotherapy routines or multidisciplinary pain management pathways.

This treatment may be especially relevant for patients reporting persistent nociceptive symptoms despite conventional rehabilitation, or in those with stress-related autonomic imbalance. While the current study supports the feasibility of a single-session approach, repeated or personalized PAPIMI applications may yield additional benefits—though future research is needed to explore dose-response relationships and optimal treatment schedules.

Clinicians should be aware that PAPIMI is not intended as a stand-alone cure but rather as a complementary modality that can enhance physiological readiness, patient comfort, and engagement with broader therapeutic interventions.

## 5. Conclusions

In conclusion, while the present findings suggest that PAPIMI therapy may represent a promising non-pharmacological approach for the acute modulation of chronic musculoskeletal pain and autonomic activity, these results should be regarded as preliminary and not yet conclusive. Further randomized, controlled, and blinded studies are needed before any inference regarding clinical applicability can be made.

## Figures and Tables

**Table 1:** Numerical Pain Rating Scale (NPRS) scores and values of heart rate variability at baseline (T0) and post intervention (T1).

Variables	T0		T1		T1 vs. T0
	Median	IQR	Median	IQR	(Z, *p*, r)
NPRS	6	4	4	3	−4.80, 0.001, −0.9
RMSSD	20.8	14.6	26.5	21.77	2.42, 0.015, 0.4
SDNN	29.1	23.09	32.6	23.52	1.70, 0.088, -
NN50	8.5	33.25	16	42	1.10, 0.274, -
PNN50	2.2	10.19	4.6	13.40	1.46, 0.144, -
HF	152.8	236.2	239.1	493.3	2.17, 0.029, 0.4

Data (n = 30) are presented as median and interquartile range (IQR = Q3 − Q1). Statistical comparisons between T1 and T0 were conducted using Wilcoxon signed-rank tests; significance level was set to alpha = 0.05; and r: estimation of the effect size with the Pearson’s correlation coefficient.

## Data Availability

The data presented in this study are available on request from the corresponding authors. The data are not publicly available due to privacy issues.
